# Family structure and multiple domains of child well-being in the United States: a cross-sectional study

**DOI:** 10.1186/s12963-015-0038-0

**Published:** 2015-02-21

**Authors:** Patrick M Krueger, Douglas P Jutte, Luisa Franzini, Irma Elo, Mark D Hayward

**Affiliations:** Department of Health and Behavioral Sciences, Campus Box 188, PO Box 173364, Denver, CO 80217-3364 USA; School of Public Health, University of California, Berkeley, USA; University of Maryland, School of Public Health, College Park, Maryland USA; Department of Sociology, University of Pennsylvania, Philadelphia, PA USA; Department of Sociology, University of Texas, Texas, USA

**Keywords:** Children, Family, Extended family, Access to health care, Utilization of health care, Health, School, Single parent, Grandparent

## Abstract

**Background:**

We examine the association between family structure and children’s health care utilization, barriers to health care access, health, and schooling and cognitive outcomes and assess whether socioeconomic status (SES) accounts for those family structure differences. We advance prior research by focusing on understudied but increasingly common family structures including single father families and five different family structures that include grandparents.

**Methods:**

Our data on United States children aged birth through 17 (unweighted N = 198,864) come from the 1997–2013 waves of the National Health Interview Survey, a nationally representative, publicly available, household-based sample. We examine 17 outcomes across nine family structures, including married couple, cohabiting couple, single mother, and single father families, with and without grandparents, and skipped-generation families that include children and grandparents but not parents. The SES measures include family income, home ownership, and parents’ or grandparents’ (depending on who is in the household) employment and education.

**Results:**

Compared to children living with married couples, children in single mother, extended single mother, and cohabiting couple families average poorer outcomes, but children in single father families sometimes average better health outcomes. The presence of grandparents in single parent, cohabiting, or married couple families does not buffer children from adverse outcomes. SES only partially explains family structure disparities in children’s well-being.

**Conclusions:**

All non-married couple family structures are associated with some adverse outcomes among children, but the degree of disadvantage varies across family structures. Efforts to understand and improve child well-being might be most effective if they recognize the increasing diversity in children’s living arrangements.

## Background

United States children increasingly live in a diverse array of family structures. Between 1970 and 2013 the share of children living with two married parents fell (by 24%), whereas the share living with single mothers has doubled (to 23.7% in 2013), the share living with single fathers has nearly quadrupled (to 4.1% in 2013), and the share living with grandparents has doubled (to 6.2% in 2013) [1]. Children’s living arrangements have been linked to their intellectual stimulation, progress through school, access to health care, hospitalizations, and unhealthy behaviors [2-8], as well as health outcomes including asthma, migraines, ear infections, allergies, obesity, and global health [9,10]. Prior research, however, focuses primarily on married couple and single mother families and seldom considers other less common but increasingly prevalent family structures.

We advance research in three ways. First, we focus on family structures that are understudied but that are increasingly common in the US, including single father, cohabiting, extended (including at least one parent and at least one grandparent), and skipped-generation (including children and grandparents, but not parents) families. Married couples are legally recognized relationships that average higher socioeconomic status (SES) and greater access to health care than other family structures [11]. Children in single mother families average worse schooling, behavioral, and health outcomes than children in married couple families [3,6,10]. The limited research on single father families is marked by inconsistent results. Some studies find few differences between single mother and single father families, but others show that children in single father families have equivalent or even superior health to those living in married couple families [9,12]. Children in cohabiting families average worse health and educational outcomes than children in married couple families, because parents who are violent or not stably employed are more likely to cohabit than to marry, and because cohabiting unions receive less social support than marriages, have fewer legal rights, and are more likely to end in dissolution [6,13-15].

Scant research examines well-being among children who live with grandparents. Grandparents who join married couple, single parent, or cohabiting families may supplement parents’ time supervising children or contribute money to the household [16]. But grandparents who are in poor health or who have few economic resources might draw money and social support away from children [17]. Grandparents who are the primary caretakers of their grandchildren because parents are absent (e.g., incarcerated, incapacitated by drugs or alcohol) could have few resources and outdated parenting skills [18-21].

Second, we provide a broad overview of the relationship between family structure and children’s well-being by examining multiple outcomes in four domains: barriers to health care, health care utilization, health, and schooling and cognitive outcomes. Children’s poor health and school performance have consequences for health and earnings throughout their lives [22,23]. Barriers to health care and inefficient health care utilization can signal the presence of unmet needs for health care, limit the effective treatment of medical conditions, and lead to elevated health care expenses for families and communities [5]. By considering outcomes in multiple domains that range from mild to severe, we will provide a more comprehensive view of the association between family structure and child well-being than prior research.

Third, we examine whether four measures of SES account for family structure disparities in children’s outcomes, given the lower SES in non-married couple families [18,24]. We focus on family income [25,26], parent and grandparent participation in the paid labor force and education levels [26,27], and home ownership, a key form of wealth for many families [28,29].

## Methods

We use publicly available data from the 1997–2013 waves of the National Health Interview Survey (NHIS), a large, cross-sectional, household-based survey that conducts face-to-face interviews with a representative sample of the non-institutionalized US population [30]. The NHIS has a response rate of 90% for eligible households. Starting in 1997, the NHIS selects a random child aged birth through 17 years (unweighted N = 198,844) from each household about whom they collect items about health, health care, and cognitive and schooling outcomes. Because some outcome variables are not included in some waves or are only meaningful for children of specific ages (noted below), the sample sizes are somewhat smaller for some analyses. The Institutional Review Board at the University of Colorado Denver approved this research.

### Variables

We examine outcome variables in four domains. Four variables indicate *barriers to health care*. The first variable is the sum of five dichotomous items that indicate reasons for delaying medical care for the child: unable to get through to the physician’s office on the phone, unable to get an appointment soon enough, the wait to see the doctor is too long, unable to get to the doctor’s office when they were open, and unable to find transportation to the doctor’s office. Three variables are dichotomous and indicate whether the child has a usual place to receive routine medical care, other than the emergency room (ER); whether the child did not receive a prescription medication in the last 12 months because of cost; and whether children aged 2 to 17 did not receive dental care in the past 12 months because of cost.

Two variables assess *health care utilization*. A dichotomous item indicates whether a child has failed to receive a well-child checkup in the last 12 months. An ordinal item indicates the number of times a child has been to the ER in the past year: none, one time, two to three times, four to nine times, 10 to 12 times, or 13 or more times.

Eight variables capture *health outcomes*. Dichotomous items indicate whether the child had frequent or severe headaches, including migraines, in the past year (ages 3 through 17); had a functional limitation that limited the child’s ability to play, remember, walk, undertake personal care, feed his or her self or that required special education or early intervention services; had a cold in the two weeks prior to interview (only available from 1998 to 2011); had three or more ear infections in the past year; had a condition for which he or she regularly took prescription medication for at least three months; or had anemia in the past year. An ordinal item indicates the presence and severity of asthma: never diagnosed with asthma, ever diagnosed with asthma but no asthma attacks in the past 12 months, ever diagnosed with asthma and had an asthma attack in the past 12 months, and ever diagnosed with asthma and had an asthma attack in the past 12 months that resulted in ER care. More severe asthma is correlated with having taken prescription medication for the past three months (polychoric correlation = 0.56), but these items offer different insights into children’s health. A final ordinal variable indicates the primary caregiver’s assessment of the child’s global health with categories including poor (=4), fair, good, very good, or excellent (=0).

Three items capture *schooling and cognitive outcomes* for children aged 6 through 17. The number of school days missed in the past school year ranges from 0 to 240. Two dichotomous variables indicate whether a school representative or health professional has ever told the caregiver that the child has a learning disability or that the child has attention deficit disorder/attention deficit hyperactivity disorder (ADD/ADHD).

Family structure is measured categorically as: married couple (referent), cohabiting couple, single mother, single father, extended married couple (including married parents and at least one grandparent), extended cohabiting couple, extended single mother, extended single father, and skipped generation (including at least one grandparent but no parents). We exclude the 0.002% of children who do not live with grandparents or parents because the NHIS provides scant information about those family structures. The NHIS does not distinguish among adopted, step, or biological children.

All models adjust for children’s demographic characteristics. Age is measured in single years and ranges from 0 to 17. We include age-squared to capture non-linear associations between age and each outcome. Sex is dichotomous. Caregiver reported race/ethnicity is categorical: non-Hispanic white (referent), non-Hispanic black, Mexican American, other Hispanic, or other non-Hispanic. A dummy variable indicates whether the child was born in the US or elsewhere. Year of survey is measured continuously and ranges from 0 (in 1997) to 17 (in 2013) to capture annual trends. Dummy variables for the calendar quarter of interview capture seasonal variation. Census region is categorical: Northeast (referent), South, Midwest, or West. A continuous variable indicates the number of children in the household. Dummy variables indicate whether children were born at very low birth weight (<1,500 grams), low birth weight (1,500 to 2,499 grams), or adequate birth weight (≥2500 grams). Caregiver recall of children’s birth weight is imperfect, but remains predictive of children’s health outcomes through age 17 [31,32]. The models for health and cognitive outcomes also adjust for whether children had a checkup in the last year because forgone checkups may result in underdiagnosis of those outcomes.

Our full models adjust for family SES. Family income was reported in categories that varied across survey years. To approximate a continuous variable, we take the midpoint of each closed-ended interval, estimate a median value for the open-ended interval, and convert all values to 2012 dollars. We adjust for the purchasing power for families of different sizes by dividing family income by family size raised to the power of 0.38, and we take the log to account for the diminishing returns to health as income increases [26,33]. The educational attainment of the most highly educated parent or grandparent, depending on who is in the household, is coded categorically as less than a high school degree, high school degree or equivalent, some college, a baccalaureate degree, and any post-baccalaureate education. Models that further adjust for the average years of education among caregivers do not improve model fit. We also include the proportion of caregivers in the household who are employed. A dichotomous item indicates whether the family owns or is purchasing their house, or is renting.

### Analysis

We use negative binomial regression to predict count outcomes (i.e., number of reasons for delaying medical care, school days missed), ordered logistic regression to model ordered outcomes (i.e., visits to the ER in the past 12 months, asthma severity, global health), and logistic regression to model the remaining binary outcomes [34,35]. Negative binomial models account for the over-dispersion of our count variables (i.e., their variances are greater than their means). Ordered logistic regression provides a single odds ratio for each predictor that summarizes the cumulative odds of having higher values on ordinal outcomes. We report exponentiated coefficients—odds ratios for logistic and ordered logistic models and incidence rate ratios for negative binomial models.

Most variables are missing from less than 3% of the observations, although family income is missing for 10%. We use multiple imputation with chained equations to impute all variables in our analyses with any missing data. Multiple imputation relies on more plausible assumptions than listwise deletion, especially when numerous variables are included in the imputation model [36-38]. Our imputation model includes over 60 variables, including items where respondents indicate whether their incomes are above or below $20,000 and above or below $50,000—items that are missing much less often than the more detailed income measure used in our analyses. All analyses account for the stratified and clustered design of the NHIS and incorporate sample weights to ensure that our results are representative of US children aged birth through 17, between 1997 and 2013.

## Results

Table 1 presents means and percentages of our variables.

**Table 1:** **Means and percentages of study variables, children aged birth through 17, United States, 1997-2013**

**Barriers to health care**		**Family structure, %**	
Num. of reasons delayed care, mean	0.14	Married couple	67
Has routine place for care, %	92	Cohabiting couple	6
No Rx because of cost, %	3	Single mother	16
No dentist due to cost (ages 2+), %	6	Single father	2
**Utilization of health care**		Extended married couple	3
No checkup last 12 mos., %	25	Extended cohabiting couple	<1
Times to the ER past 12 mos., %		Extended single mother	4
None	80	Extended single father	1
1 visit	13	Skipped generation	1
2-3 visits	5	**Family and caregiver SES**	
4-9 visits	1	Family income equivalence, mean	$42,751
10-12 visits	<1	Highest educational attainment, %	
13+ visits	<1	Less than high school	10
**Health outcomes**		High school degree	24
Global health, %		Some college	32
Excellent	56	Baccalaureate degree	20
Very good	27	Post-baccalaureate	14
Good	15	Proportion employed, %	74
Fair	2	Homeowner, %	66
Poor	<1	**Children’s demographic characteristics**	
Headaches last 12 mos. (ages 3+), %	6	Age, mean	8.5
Activity limitation, %	8	Male, %	51
Cold last 2 weeks, 1998–2013, %	18	Race/Ethnicity, %	
3+ ear infections last 12 mo., %	6	Non-Hispanic white	61
Condition requiring Rx for 3+ mos., %	13	Non-Hispanic black	15
Asthma severity, %		Non-Hispanic other	6
Never diagnosed with asthma	87	Mexican American	13
Diagnosed but no attacks in 12 mos.	7	Other Hispanic	6
Diagnosed & attack in 12 mos.	4	Foreign born, %	4
Diagnosed & hospitalized 12 mos.	2	Survey year, mean	2005
Anemia last 12 mos., %	1	Region of residence, %	
**Schooling and cognitive outcomes (ages 6+)**		Northeast	17
School days missed last year, mean	4	South	36
Learning disability, %	9	Midwest	24
ADD/ADHD, %	8	West	22
		Number children in family, mean	2.4
		Calendar quarter of interview, %	
		First quarter	25
		Second quarter	25
		Third quarter	25
		Fourth quarter	25
		Birth weight, %	
		≥2,500 grams	90
		1,500 to 2,499 grams	7
		<1,500 grams	3
N (unweighted): 198,894			

### Barriers to health care and health care utilization

Table 2 presents results for the association between family structure and health care outcomes. Panel A shows models that adjust for demographic variables. Children in cohabiting, single mother, skipped generation, and most extended families delay care for 1.19 to 1.78 times as many reasons as children in married couple families, but children in single father and extended single father families do not delay care for more reasons. Compared to children in married couple families, children in all other family structures have lower odds of having a routine place for care, and children in all other family structures except skipped generation families have higher odds of forgoing prescription medications and dental care due to cost. Results for health care utilization show that children in all but extended married couple and extended single mother families have higher odds of going without a well-child checkup than children in married couple families, and children in all non-married couple families have higher odds of going to the ER more times.

**Table 2:** **Exponentiated coefficients for health care outcomes, children aged birth through 17, United States, 1997–2013**

	**Barriers to health care**				**Health care utilization**	
	**Reasons for delay** ^**e**^	**Routine place for care** ^**c**^	**No Rx due to cost** ^**c**^	**No dental care due to cost** ^**c**^	**No well-child checkup** ^**c**^	**Times ER** ^**d**^
	**Panel A: Reduced model** ^**a**^					
Married couple	ref.	ref.	ref.	ref.	ref.	ref.
Cohabiting couple	1.58***	0.67***	1.97***	1.82***	1.22***	1.60***
	(1.44,1.74)	(0.62,0.73)	(1.72,2.27)	(1.64,2.02)	(1.15,1.30)	(1.52,1.70)
Single mother	1.76***	0.83***	2.25***	1.86***	1.06**	1.70***
	(1.67,1.86)	(0.78,0.88)	(2.07,2.45)	(1.75,1.99)	(1.02,1.10)	(1.63,1.77)
Single father	0.96	0.53***	1.38**	1.27**	1.43***	1.17**
	(0.82,1.13)	(0.47,0.60)	(1.10,1.73)	(1.10,1.48)	(1.31,1.56)	(1.06,1.28)
Extended married couple	1.28***	0.87*	1.63***	1.40***	1.08	1.23***
	(1.14,1.43)	(0.76,0.98)	(1.35,1.98)	(1.17,1.67)	(0.99,1.17)	(1.13,1.34)
Extended cohabiting couple	1.78***	0.72*	2.71***	2.62***	1.30*	1.83***
	(1.33,2.40)	(0.54,0.97)	(1.79,4.12)	(1.81,3.79)	(1.01,1.67)	(1.48,2.27)
Extended single mother	1.58***	0.85**	2.21***	1.38***	1.05	1.73***
	(1.43,1.75)	(0.76,0.95)	(1.91,2.57)	(1.21,1.58)	(0.97,1.13)	(1.62,1.86)
Extended single father	0.94	0.59***	1.89***	1.46**	1.23*	1.44***
	(0.72,1.22)	(0.48,0.72)	(1.33,2.70)	(1.10,1.93)	(1.05,1.44)	(1.23,1.67)
Skipped generation	1.19*	0.76***	0.98	1.03	1.13*	1.49***
	(1.01,1.40)	(0.66,0.88)	(0.76,1.26)	(0.84,1.25)	(1.03,1.26)	(1.33,1.67)
	**Panel B: Full model** ^**b**^					
Married couple	ref.	ref.	ref.	ref.	ref.	ref.
Cohabiting couple	1.30***	0.87***	1.33***	1.30***	1.02	1.32***
	(1.18,1.43)	(0.80,0.94)	(1.16,1.53)	(1.17,1.45)	(0.96,1.09)	(1.25,1.40)
Single mother	1.30***	1.23***	1.23***	1.13**	0.83***	1.31***
	(1.22,1.38)	(1.15,1.31)	(1.13,1.35)	(1.05,1.21)	(0.79,0.87)	(1.26,1.37)
Single father	0.84*	0.68***	0.97	0.94	1.18***	1.00
	(0.71,0.99)	(0.60,0.77)	(0.77,1.23)	(0.81,1.10)	(1.08,1.28)	(0.91,1.11)
Extended married couple	1.25***	0.84**	1.66***	1.41***	1.07	1.21***
	(1.12,1.40)	(0.74,0.96)	(1.37,2.02)	(1.18,1.69)	(0.98,1.17)	(1.11,1.32)
Extended cohabiting couple	1.62**	0.83	2.23***	2.22***	1.13	1.62***
	(1.21,2.16)	(0.61,1.11)	(1.47,3.39)	(1.53,3.22)	(0.88,1.45)	(1.31,2.01)
Extended single mother	1.35***	1.01	1.64***	1.06	0.91*	1.50***
	(1.22,1.50)	(0.90,1.13)	(1.41,1.91)	(0.92,1.22)	(0.84,0.99)	(1.40,1.61)
Extended single father	0.81	0.72**	1.38	1.10	1.02	1.24**
	(0.61,1.08)	(0.59,0.89)	(0.96,1.97)	(0.83,1.46)	(0.87,1.20)	(1.06,1.44)
Skipped generation	0.90	1.09	0.62***	0.72**	0.86**	1.22**
	(0.76,1.06)	(0.94,1.27)	(0.48,0.81)	(0.59,0.88)	(0.77,0.95)	(1.08,1.37)
N (unweighted)	198,894	198,894	198,894	174,723	198,894	198,894

*P < 0.05; ** < 0.01; ***P < 0.001 (2-tailed tests).

^a^The models in Panel A adjust for children’s characteristics.

^b^The models in Panel B adjust for family SES in addition to the covariates included in the models in Panel A. The models in Panel B fit better than the respective models in Panel A at the P < 0.001 level in all cases.

^c^This column presents odds ratios from a logistic regression model.

^d^This column presents odds ratios from an ordered logistic regression model.

^e^This column presents incidence rate ratios from a negative binomial regression model.

Panel B shows that family structure disparities persist when adjusting for SES. Children in cohabiting couple and extended married couple families remain more likely to experience all four barriers to care than children in married couple families, and the number of reasons for delaying care and the odds of forgoing prescription medications or dental care due to cost remain elevated among children in single mother and extended cohabiting couple families. Nevertheless, when adjusting for SES, the exponentiated coefficients in Panel B are attenuated (i.e., closer to 1) compared to those in Panel A. Indeed, children in skipped generation families are no different than those in married couple families in the number of reasons for delaying care or access to a routine place for care, and have lower odds of forgoing dental care or prescription medications due to cost. Further, children in single mother families have 23% higher odds of having a routine place for care, and children in single father families have 16% fewer reasons for delaying care than children in married couple families.

In terms of health care utilization, only children in single father families have higher odds of forgoing a well-child checkup than children in married couple families, after adjusting for SES, and children in single mother, extended single mother, and skipped generation families have lower odds of forgoing a checkup. But children in all non-married couple families, except single father families, have elevated odds of going to the ER more times.

### Health, schooling, and cognitive outcomes

Table 3 presents results for the association between family structure and health, schooling, and cognitive outcomes. Panel A shows results from models that adjust for the demographic variables, and for all outcomes except days missed from school, whether children had a checkup in the last year. Compared to children in married couple families, children in all other family structures have higher odds of having worse global health.

**Table 3:** **Exponentiated coefficients for health, schooling, and cognitive outcomes, children aged birth through 17, United States, 1997–2013**

	**Health outcomes**								**Schooling and cognitive outcomes**		
	**Global health** ^**d**^	**Headache** ^**c**^	**Activity limitation** ^**c**^	**Cold** ^**c**^	**Ear infection** ^**c**^	**Requiried Rx for 3+ mos.** ^**c**^	**Asthma severity** ^**d**^	**Anemia** ^**c**^	**School days missed** ^**e**^	**Learning disability** ^**c**^	**ADD/ADHD** ^**c**^
	**Panel A: Reduced model** ^**a**^										
Married couple	ref.	ref.	ref.	ref.	ref.	ref.	ref.	ref.	ref.	ref.	ref.
Cohabiting couple	1.52***	1.55***	1.51***	1.05	1.20***	1.18***	1.31***	1.43***	1.23***	1.78***	1.93***
	(1.44,1.59)	(1.38,1.74)	(1.37,1.66)	(0.98,1.13)	(1.08,1.33)	(1.09,1.28)	(1.21,1.43)	(1.16,1.76)	(1.11,1.35)	(1.58,2.01)	(1.72,2.16)
Single mother	1.54***	1.57***	1.82***	1.20***	1.43***	1.42***	1.50***	1.64***	1.48***	1.76***	1.74***
	(1.49,1.59)	(1.47,1.68)	(1.72,1.92)	(1.15,1.26)	(1.34,1.53)	(1.35,1.48)	(1.44,1.57)	(1.42,1.89)	(1.40,1.57)	(1.65,1.88)	(1.63,1.85)
Single father	1.11**	0.89	1.12	0.85**	0.85	0.72***	0.91	0.70	0.96	1.26**	1.31***
	(1.03,1.19)	(0.76,1.06)	(0.98,1.29)	(0.75,0.96)	(0.68,1.08)	(0.64,0.80)	(0.81,1.02)	(0.45,1.10)	(0.87,1.05)	(1.08,1.47)	(1.15,1.51)
Extended married couple	1.36***	1.24*	1.25**	1.03	1.11	1.11	1.15*	1.32*	1.36**	1.33**	1.36**
	(1.26,1.45)	(1.04,1.48)	(1.08,1.43)	(0.94,1.14)	(0.96,1.27)	(0.98,1.25)	(1.02,1.29)	(1.01,1.72)	(1.12,1.64)	(1.09,1.63)	(1.10,1.68)
Extended cohabiting couple	1.58***	1.54	1.70**	0.89	1.25	0.92	1.30	1.61	1.43	2.40***	1.97*
	(1.31,1.90)	(0.86,2.74)	(1.14,2.53)	(0.70,1.14)	(0.89,1.76)	(0.63,1.35)	(0.95,1.77)	(0.75,3.47)	(0.96,2.13)	(1.51,3.81)	(1.13,3.45)
Extended single mother	1.85***	1.82***	1.52***	1.19***	1.36***	1.35***	1.60***	1.39**	1.59***	1.66***	1.54***
	(1.74,1.96)	(1.57,2.10)	(1.34,1.72)	(1.10,1.29)	(1.22,1.51)	(1.24,1.49)	(1.46,1.76)	(1.14,1.71)	(1.40,1.81)	(1.44,1.92)	(1.34,1.77)
Extended single father	1.85***	1.41*	1.46**	1.10	1.20	1.03	1.14	1.50	1.08	1.43**	1.74***
	(1.60,2.13)	(1.07,1.85)	(1.14,1.87)	(0.91,1.33)	(0.91,1.59)	(0.84,1.27)	(0.93,1.41)	(0.86,2.61)	(0.91,1.28)	(1.09,1.87)	(1.34,2.27)
Skipped generation	1.85***	1.39***	2.03***	1.13	1.33**	1.52***	1.48***	1.37	1.34***	2.16***	2.32***
	(1.68,2.04)	(1.17,1.65)	(1.75,2.35)	(1.00,1.29)	(1.10,1.60)	(1.34,1.73)	(1.32,1.66)	(0.95,1.98)	(1.18,1.52)	(1.82,2.55)	(1.99,2.70)
	**Panel B: Full model** ^**b**^										
Married couple	ref.	ref.	ref.	ref.	ref.	ref.	ref.	ref.	ref.	ref.	ref.
Cohabiting couple	1.14***	1.26***	1.25***	1.04	1.10	1.15**	1.19***	1.13	1.09	1.43***	1.61***
	(1.08,1.20)	(1.12,1.42)	(1.13,1.38)	(0.97,1.12)	(0.98,1.22)	(1.05,1.24)	(1.09,1.29)	(0.91,1.40)	(0.97,1.22)	(1.26,1.63)	(1.42,1.81)
Single mother	1.02	1.15***	1.36***	1.16***	1.26***	1.31***	1.32***	1.16	1.20***	1.26***	1.38***
	(0.98,1.05)	(1.07,1.25)	(1.28,1.45)	(1.10,1.22)	(1.17,1.36)	(1.25,1.38)	(1.25,1.38)	(0.99,1.37)	(1.12,1.28)	(1.18,1.36)	(1.28,1.48)
Single father	0.85***	0.75**	0.97	0.84**	0.79*	0.72***	0.85**	0.59*	0.87**	1.05	1.16*
	(0.79,0.91)	(0.64,0.89)	(0.85,1.12)	(0.75,0.95)	(0.63,0.99)	(0.64,0.81)	(0.76,0.96)	(0.38,0.93)	(0.80,0.96)	(0.89,1.22)	(1.01,1.34)
Extended married couple	1.32***	1.16	1.12	1.03	1.09	1.04	1.12	1.28	1.27*	1.18	1.23
	(1.23,1.41)	(0.97,1.39)	(0.97,1.29)	(0.93,1.14)	(0.95,1.25)	(0.92,1.18)	(0.99,1.25)	(0.98,1.68)	(1.06,1.53)	(0.97,1.44)	(1.00,1.52)
Extended cohabiting couple	1.26*	1.28	1.39	0.89	1.17	0.88	1.20	1.38	1.27	1.88**	1.63
	(1.05,1.52)	(0.71,2.29)	(0.93,2.06)	(0.69,1.14)	(0.83,1.64)	(0.60,1.29)	(0.88,1.65)	(0.64,2.98)	(0.82,1.96)	(1.18,2.99)	(0.93,2.86)
Extended single mother	1.45***	1.43***	1.17*	1.16***	1.26***	1.23***	1.46***	1.14	1.33***	1.22**	1.24**
	(1.36,1.53)	(1.24,1.66)	(1.03,1.33)	(1.07,1.26)	(1.13,1.40)	(1.12,1.36)	(1.33,1.60)	(0.92,1.40)	(1.16,1.51)	(1.06,1.42)	(1.07,1.43)
Extended single father	1.38***	1.07	1.08	1.08	1.10	0.94	1.04	1.20	0.89	0.99	1.38*
	(1.19,1.59)	(0.82,1.42)	(0.84,1.40)	(0.89,1.31)	(0.84,1.46)	(0.77,1.16)	(0.84,1.28)	(0.69,2.11)	(0.75,1.06)	(0.76,1.30)	(1.06,1.79)
Skipped generation	1.19***	0.99	1.40***	1.10	1.17	1.38***	1.33***	0.99	1.02	1.37***	1.78***
	(1.08,1.31)	(0.83,1.18)	(1.20,1.63)	(0.97,1.25)	(0.97,1.42)	(1.21,1.58)	(1.18,1.50)	(0.67,1.45)	(0.90,1.16)	(1.15,1.64)	(1.51,2.09)
N (unweighted)	198,894	162,853	198,894	171,892	198,894	198,894	198,894	198,894	129,687	129,687	129,687

*P < 0.05; ** < 0.01; ***P < 0.001 (2-tailed tests).

^a^The models in Panel A adjust for children’s demographic characteristics. All of the models, except for the model for the number of school days missed, also adjust for whether the child had a checkup in the past year.

^b^The models in Panel B adjust for family SES in addition to the covariates included in the models in Panel A. The models in Panel B fit better than the respective models in Panel A at the P < 0.001 level in all cases.

^c^This column presents odds ratios from a logistic regression model.

^d^This column presents odds ratios from an ordered logistic regression model.

^e^This column presents incidence rate ratios from a negative binomial regression model.

Of the remaining health outcomes, children in single mother and extended single mother families have higher odds of all seven, children in cohabiting couple families have higher odds of six, children in skipped generation families have higher odds of five, children living in extended married couple families have higher odds of four, children in extended single father families have higher odds of two, and children in extended cohabiting families have higher odds of one, compared to children in married couple families. Children in single father families have *lower* odds of having the common cold or a medical condition that requires prescription medication for three or more months and similar odds of having the other five outcomes, compared to children in married couple families.

Results for the schooling and cognitive outcomes show that school-aged children in cohabiting, single mother, extended married couple, extended single mother, and skipped generation families miss 1.23 to 1.59 times as many days of school per year as children in married couple families. Living in any non-married couple family is associated with higher odds of having a learning disability or ADD/ADHD.

Panel B shows that, after adjusting for SES, the non-married couple family structures remain associated with worse global health, except for children in single mother families who are no different from children in married couple families, and children in single father families who have 15% lower odds of being in worse health. Family structure disparities in other outcomes persist but are attenuated. Notably, when adjusting for SES, children in extended married couple, extended cohabiting couple, and extended single father families are not disadvantaged on any of the seven remaining health outcomes, and children in single father families are *advantaged* on six of the outcomes. Adjusting for SES also partially accounts for the association between family structure and schooling and cognitive outcomes.

## Discussion

We advance research by examining nine family structures that reflect children’s diverse living arrangements at the beginning of the 21^st^ century [1]. We find important family structure disparities in multiple domains of child well-being. In general, children in non-married couple families average worse outcomes than children in married couple families.

Upon closer examination, however, children are not equally disadvantaged across all family structures. First, consistent with prior research [3,6,10,21], single mother and extended single mother families fare worse than children in married couple families for almost all of the outcomes examined. Single mothers are generally younger, lack the benefit of a second parent, and have less social support than caregivers in other family structures [3,6,10]. Second, parents’ marital status matters. Children in cohabiting couples have worse outcomes than children in married couple families, even after adjusting for SES, perhaps because those unions often arise from the selection of less capable parents into cohabitation and lack the legal recognition of marriage [11,13]. Cohabiting parents are generally younger, less prepared for parenting, and more likely to separate than married parents—factors that may result in worse outcomes for children in those unions [6,13-15]. Third, the presence of grandparents in extended families does not mitigate the negative outcomes among children who live in single parent or cohabiting families. Perhaps because sickly or penurious grandparents draw parents’ social or economic support away from grandchildren [2,18], we find that children who live in extended married couple families have worse outcomes than children who live in married couple families.

Fourth, children in single father and, to a lesser extent, extended single father families are often less disadvantaged than children in single mother or extended single mother families, respectively. After adjusting for SES, children in single father families have lower odds of worse global health, were advantaged on six other health outcomes, and missed fewer days of school than children in married couple families. The relatively advantaged health for children in single father families may reflect differences in how mothers and fathers report child outcomes [39]. Or, single mothers and single fathers may differ in their knowledge about their children’s health; compared to children of single mothers, children of single fathers are less likely to have a routine place to receive medical care, and are more likely to have forgone a well-child checkup in the last 12 months. Alternately, mothers and fathers may select into single parenting in different ways—single fathers are generally older and more likely to be previously married than single mothers, factors that may be associated with better outcomes for children. Additional research is necessary to understand the variable associations between single father families and child outcomes reported in our analyses and in prior studies [9,12,14].

Finally, children in skipped generation families have worse health, schooling, and cognitive outcomes than children in married couple families, even after adjusting for SES. Grandparents may be caregivers of last resort when both parents are absent [18-21]. But SES adjusted models show that children in skipped generation families are relatively advantaged on two of the barriers to care outcomes and both of the health care utilization variables.

In general, SES attenuates the association between family structure and children’s well-being. For every outcome examined, the relationship between family structure and children’s well-being was weakened, sometimes fully explained, and occasionally reversed once adjusting for family income, caregivers’ education and employment, and home ownership [10,18,21,25]. But SES could only completely explain the association between family structure and anemia, and many family structure disparities remain [2,7]. Future research could explore other potential mediators and confounders of the association between family structure and children’s well-being.

### Limitations

Four limitations of our study warrant mention. First, all of our outcome variables were reported by caregivers, although research suggests that caregivers’ reports of children’s health are valid [31,32,40]. Second, our data are cross-sectional and cannot demonstrate causal associations between family structure and children’s outcomes. Nevertheless, our results describe groups of children who may warrant additional attention in health care and school settings. Third, our data offer no insight into children’s interactions with non-residential parents [12]. Finally, although our data include multiple measures of socioeconomic status, our data do not include measures of the value of assets or caregivers’ occupational statuses. Additional measures of SES may further explain family structure disparities in child outcomes.

## Conclusion

US children increasingly live in family structures that are associated with poor child well-being. The links between childhood circumstances and socioeconomic and health outcomes in later life mean that children’s disadvantages may persist throughout their lives [22,41]. Growing diversity in US family structures suggests the need to better understand the associations between children’s well-being and their family contexts.

## Abbreviation

SES: Socioeconomic status
